# 

**Published:** 1870-09

**Authors:** 


### BOOK NOTICE.

"Maternity; A Treatise for Wives and Mothers," published by J. B. Ford & Co., 39 Park Row, New York. $2.25. Author, Dr. Verdi of Washington City. It abounds with most valuable information and should be owned by every intelligent mother.

